# Stereotactic body radiotherapy for recurrent and oligometastatic soft tissue sarcoma

**DOI:** 10.1186/s12957-022-02781-1

**Published:** 2022-09-29

**Authors:** Xiao-Yao Feng, Jing Li, Ao-Mei Li, Sheng-Hua Jing, Xi-Xu Zhu, Zhen Wang

**Affiliations:** Department of Radiation Oncology, Affiliated Jinling Hospital, Medical School of Nanjing University, Nanjing, 210002 Jiangsu China

**Keywords:** Stereotactic body radiotherapy (SBRT), Soft tissue sarcoma (STS), Oligometastatic, Radiotherapy, Recurrent

## Abstract

**Background:**

Soft tissue sarcoma (STS) is a malignant tumor of highly heterogeneous mesenchymal origin. STS has a biological pattern and clinical transformation with localized invasive growth and is susceptible to hematogenous metastasis. Local therapeutic strategies may treat recurrent and oligometastatic STS, including surgery and radiation therapy. This study aimed to evaluate the safety and efficacy of stereotactic body radiotherapy (SBRT) for recurrent and oligometastatic STS.

**Methods:**

We retrospectively analyzed 37 recurrent and oligometastatic STS patients with 58 lesions treated with SBRT from 2009 to 2019 at our institution. Oligometastatic is defined as metastatic lesions less than or equal to 3. The primary endpoint was local control (LC); secondary endpoints were survival and toxicity.

**Results:**

The median follow-up was 21.0 months (3.0 to 125.0 months). Among 37 patients, 18 were recurrent patients, and 19 were oligometastatic patients. Median LC was 25.0 months (95% CI 20.0–45.0). The 1-, 2-, and 3-year LC rates were 80.2%, 58.3%, and 46.6%, respectively. Median overall survival (OS) was 24.0 months (95% CI 13.0–28.0), and the survival rates after SBRT were 71.5%, 40.0%, and 29.1% at 1, 2, and 3-year, respectively. Median progression-free survival (PFS) was 10.0 months (95% CI 8.0–15.0 months), PFS rate after SBRT was 43.6%, 26.8%, and 18.4% at 1, 2, and 3 years, respectively. Late grade 3 radiation dermatitis was observed in one patient (2.7%). Using univariate and multivariate COX analysis, better OS, PFS, and LC were obtained in the histologic grade 1(G1) group, and tumor size and a number of lesions influenced LC.

**Conclusions:**

SBRT is a safe and effective treatment for patients with recurrent and oligometastatic STS. Histological grade influences local control and survival. SBRT may be a promising treatment option for recurrent and oligometastatic STS.

## Introduction

Sarcomas are a rare and heterogeneous group of tumors, a general term for a class of solid mesenchymal tumors. In 2021, it was estimated that in the USA 13,460 people were diagnosed with STS and 5350 died [1]. STS can occur anywhere in the body, most often in the extremities and trunk, and its local behavior is mainly longitudinal extension along the muscle cavity rather than direct invasion. For 30 years, extended local resection and postoperative radiotherapy have remained the standard treatment for STS. Most patients with soft tissue sarcoma die from tumor metastasis-related diseases. The most common site of distant metastases is the lung, followed by bone, liver, and brain, and then visceral, retroperitoneal, and other soft tissues [2]. Treatment of recurrence and metastasis is still challenging, and radiotherapy may be an effective alternative strategy for patients with difficult surgical resection or surgery resulting in serious complications [3–5].

SBRT uses rigid fixation, advanced image guidance, and complex treatment planning and delivery systems, resulting in a highly conformal dose distribution that reduces treatment volume relative to conventional radiotherapy. This, in turn, allows each fraction to deliver large doses of radiation and increase the BED compared to the traditional treatments [6]. It plays an important role in treating oligometastases tumors [7]. An increasing number of trials have reported their findings on SBRT in patients with STS suffering from lung metastasis [8–11]. Tetta C et al. [12] showed that the 2-year LC for STS patients ranged from 85 to 97%, with an OS of 47.6 months for patients who underwent SBRT, comparable to the OS of 46.7 months for patients who underwent resection of metastatic tumors. In addition, several retrospective studies on recurrent STS SBRT have been published, with promising results in terms of LC, toxicity, and OS [13].

Thus, theoretically, SBRT may be an attractive alternative to surgical resection, conventional radiotherapy or other palliative measures in certain patients with metastatic or recurrent soft tissue sarcoma. In the current study, we report our early institutional experience with SBRT in patients with recurrent and oligometastatic STS.

## Methods and materials

### Patients

We retrospectively searched our patient database for patients who received SBRT at the Radiotherapy Center of Jinling Hospital between December 2009 and December 2019. Inclusion criteria included the diagnosis of STS; experienced recurrence (defined as tumor recurrence at a site previously treated for a STS) after surgery or oligometastatic (defined as a condition characterized by a progression in a maximum of 3 metastatic sites). Exclusion criteria included patients with multiple metastases. This study was approved by the ethics committee of hospital. Medical records have been assessed for eligibility.

### Stereotactic body radiotherapy

SBRT used the Cyber-Knife Radiation Therapy System (CyberKnife®, Accuray, Sunnyvale, CA, USA). The CK can use the human skeleton, gold fiducials as a reference and monitor the target areas of the patient’s body in real time during treatment, for moving organs (such as the lung, liver, and kidney areas). Six-dimensional cranial tracking is used for intracranial, head, and neck tumors, while the “X-ray spine” tracking method is used for spinal metastases. Moving organs can implant one to three gold fiducials inside or near the tumor. One week after fiducial placement, CT simulation was performed for treatment planning (Brilliance TM Big Bore, Philips, Netherlands).

The PTV was obtained by isotropic expansion of 2–7 mm around the macroscopic lesion (bulk tumor volume, GTV) outlined on a 1.0 mm thick simulated CT scan. The dose was opened to the outer line of the PTV and 75–85% isodose line. We gave the prescription dose according to the grading and the location of the lesion. The total dose of SBRT ranged from 30 to 75 Gy over a period of 2 to 10 days. A linear-quadratic model was used for dose equivalence, assuming α/β = 10 Gy for the tumor. The median BED was 85.5 Gy, ranging from 51.3 to 187.5 Gy. Treatment planning was based on each patient’s status, tumor size, pathological type, and lesion’s location. Dosimetric indices for the 58 lesions during SBRT are listed in Table 1.

**Table 1:** Patient and tumor characteristics

Patient-related variables (*n* = 37)	Patients, *n* (%)
Age: median (range)	58 years(19–82 years)
Gender	
Male	25(67.6%)
Female	12(32.4%)
Pathological type	
Smooth muscle tumors	5(13.5%)
Fibroblastic and myofibroblastic tumors^a^	11(29.7.4%)
Tumors of uncertain differentiation^b^	8(21.6%)
Adipocytic tumors^c^	5(13.5%)
Vascular tumors	3(8.1%)
Skeletal muscle tumors	3(8.1%)
Others	2(5.4%)
Disease setting	
Recurrence	18(48.6%)
Oligometastasis	19(51.4%)
Histological grade*	
G1	19(51.4%)
G2	4(10.8%)
G3	14(37.8%)
Number of lesions^d^	
1	24(64.8%)
2–3	13(35.1%)
Systemic treatment	
Chemotherapy	18(48.6%)
Targeted therapy	4(10.8%)
Immunotherapy	1(2.7%)
Interventional therapy	2(5.4%)
None	12(32.4%)

^a^11 cases of fibroblastic and myofibroblastic tumors including 5 cases of myxofibrosarcoma, 2 cases of fibrosarcoma NOS, 1 case of inflammatory myofibroblastic tumor, 1 case of solitary fibrous tumor, malignant, 1 case of desmoid-type fibromatosis, and 1 dermatofibrosarcoma protuberans

^b^8 cases of tumors of uncertain differentiation including 3 cases of undifferentiated sarcoma, 1 case of spindle cell sarcoma, undifferentiated, 1 cases of pleural mesothelial sarcoma, 1 case of endometrial stromal sarcoma, 1 case of alveolar soft part sarcoma, 1 case of synovial sarcoma

^c^5 cases of adipocytic tumors including 3 cases of dedifferentiated liposarcoma, 2 cases of myxoid liposarcoma

^d^Number of concurrent lesions at the time of first SBRT

*Histological grade was based on the pathological findings of the primary site at the time of initial diagnosis

### Follow-up (FU) and definition of the endpoints

Efficacy was assessed using the revised solid tumor response assessment criteria (RECIST version 1.1, 2009) [14]. Clinical assessment by physical examination and CT or MRI scan was performed at 3 and 6 months, followed by every 6 months until 5 years post-treatment or until disease progression.

Local failure (LF) was defined as a minimum 20% increase in the diameter of the treated volume. The local control rate was calculated for 58 lesions from the start of radiotherapy to LF or last follow-up. OS was measured from the start of radiotherapy to death from any cause or the last follow-up visit. The PFS was calculated from the start of radiotherapy until disease progression or death. Acute toxicity and late toxicity (within 3 months or after the end of treatment, respectively) were assessed using the Common Terminology Criteria for Adverse Events (CTCAE version 4.03, 2010).

### Statistical analysis

The LC and OS curves were estimated by Kaplan-Meier analysis and were compared using the log-rank test. The influence of variables on LC and survival was investigated using univariate analysis (Cox model). These significant variables in univariate analysis and covariates considered clinically influential were then analyzed by multivariate cox regression to identify significant variables. Cox proportional hazard survival regression was used for the multivariable analyses of histological grade association with LC outcomes. Histological grade lesions size and disease setting (recurrent and oligometastatic) were selected for the models. For all analyses, two-sided tests of significance were used with *P* values < 0.05 considered significant. Statistical analysis was performed using SPSS software, version 22.0 (SPSS Inc., Chicago, IL, USA) and MedCalc software, version 19.6.

## Result

### Patient- and treatment-related characteristics

**Fig. 1:**
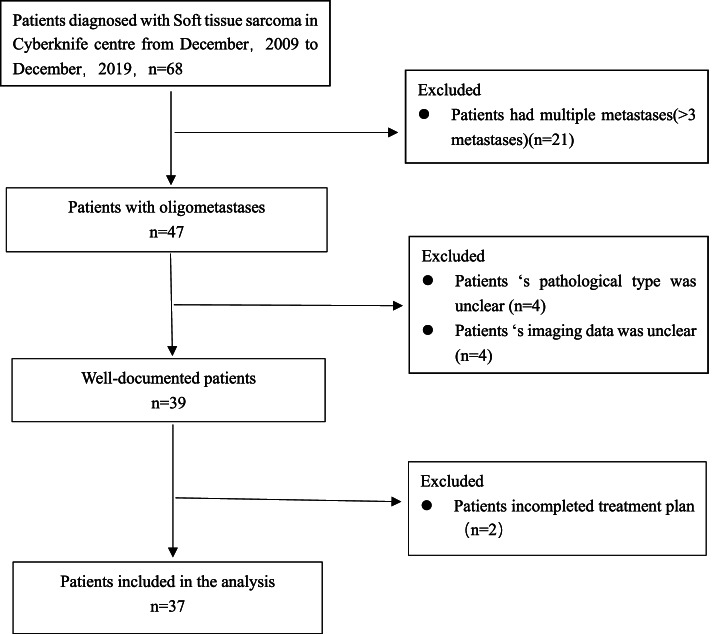
Study flow chart

We reviewed a total of 68 patients with recurrent and oligometastatic who were treated with SBRT between December 2009 and December 2019 at the Radiotherapy Center of Jinling Hospital. We excluded patients with multiple metastases, unclear pathological type or imaging data and incomplete treatment plans. Finally, 37 patients who could not or refused surgery due to difficulty of reoperation, low patient willingness, and underlying diseases with 58 lesions who completed SBRT were enrolled in our study (Fig. 1).

Patient characteristics are listed in Table 1. Of 37 patients, 18 patients were recurrence and 19 patients were oligometastatic, 31 patients presented with recurrence or oligometastatic before SBRT. The median time from diagnosis to recurrence/oligometastatic was 7 months (range 2–100 months), and 28 underwent surgery for the first treatment, 7 of whom underwent more than 2 operations. Thirteen patients received postoperative adjuvant chemotherapy and 3 patients received postoperative adjuvant radiotherapy. Main histological type were fibroblastic and myofibroblastic tumors (29.7%, 11/37), tumors of uncertain differentiation (21.6%, 8/37), and adipocytic tumors (13.5%, 5/37). 48.6%(18/37) of patients received adjuvant chemotherapy, 11 of whom were oligometastatic and 7 of whom were recurrent. Chemotherapy was mainly sequential, including 9 cases before SBRT, and 5 cases after SBRT and the remaining 4 cases were treated with concurrent chemotherapy. The main sites for 58 lesions SBRT treatments were the abdomen or pelvis (18/58, 31%), followed by the lung (17/58, 30 %). The median lesion size was 5.5 cm (1.6–20 cm), of which ≤ 5 cm accounted for 45% (26/58), and the median BED was 85.5 Gy (range 51.3–187.5 Gy). A total of 32 large-size lesions (> 5 cm) were mainly abdomen/pelvis (17/32) and lung/chest (12/32). The main treatment-related features are listed in Table 2. Since not every metastasis was biopsied, a histological grade was based on the pathological findings of the primary site at the time of initial diagnosis.

**Table 2:** Treatment-related characteristics

Treatment-related variables (*n* = 58)	Treatments, *n* (%)
Site	
Head and neck	6(10.3%)
Lung	17(29.3%)
Chest	5(8.6%)
Abdomen/pelvis	18(31.0%)
Vertebral body bone	10(17.2%)
Limbs	2(3.4%)
Lesions size: median (range)	5.5 cm (1.6–20 cm)
<5.0 cm	26(44.8%)
≥ 5.0 cm	32(55.2%)
BED: median (range)	85.5 Gy (51.3–187.5 Gy)
< 100 Gy	31(53.4%)
≥ 100 Gy	27(46.6%)
Prescription dose (Gy)/fraction	45 Gy(30–75)/5 Fx(2–10 Fx)
Median (range)	
30 Gy/3 Fx	8
45–48 Gy/3–4 Fx	4
54–60 Gy/3 Fx	3
40 Gy/5 Fx	5
45 Gy/5 Fx	11
50 Gy/5 Fx	15
Others	12
Isodose (%): median (range)	75% (55–88%)
CI: median (range)	1.13(1.00–2.77)
nCI: median (range)	1.42(1.13–2.93)
HI: median (range)	1.33(1.10–2.00)
PTV coverage: median (range)	86.12% (47.81–99.75%)

*Abbreviations*: *BED* biologically equivalent dose, *BED* values calculate using α/β = 10. *Coverage* the coverage is volume of the tumor receiving greater than or equal to the prescription dose divided by the total volume of the tumor times 100; *CI* conformity index, *nCI* new conformity index, *HI* homogeneity index

### Local control

**Fig. 2:**
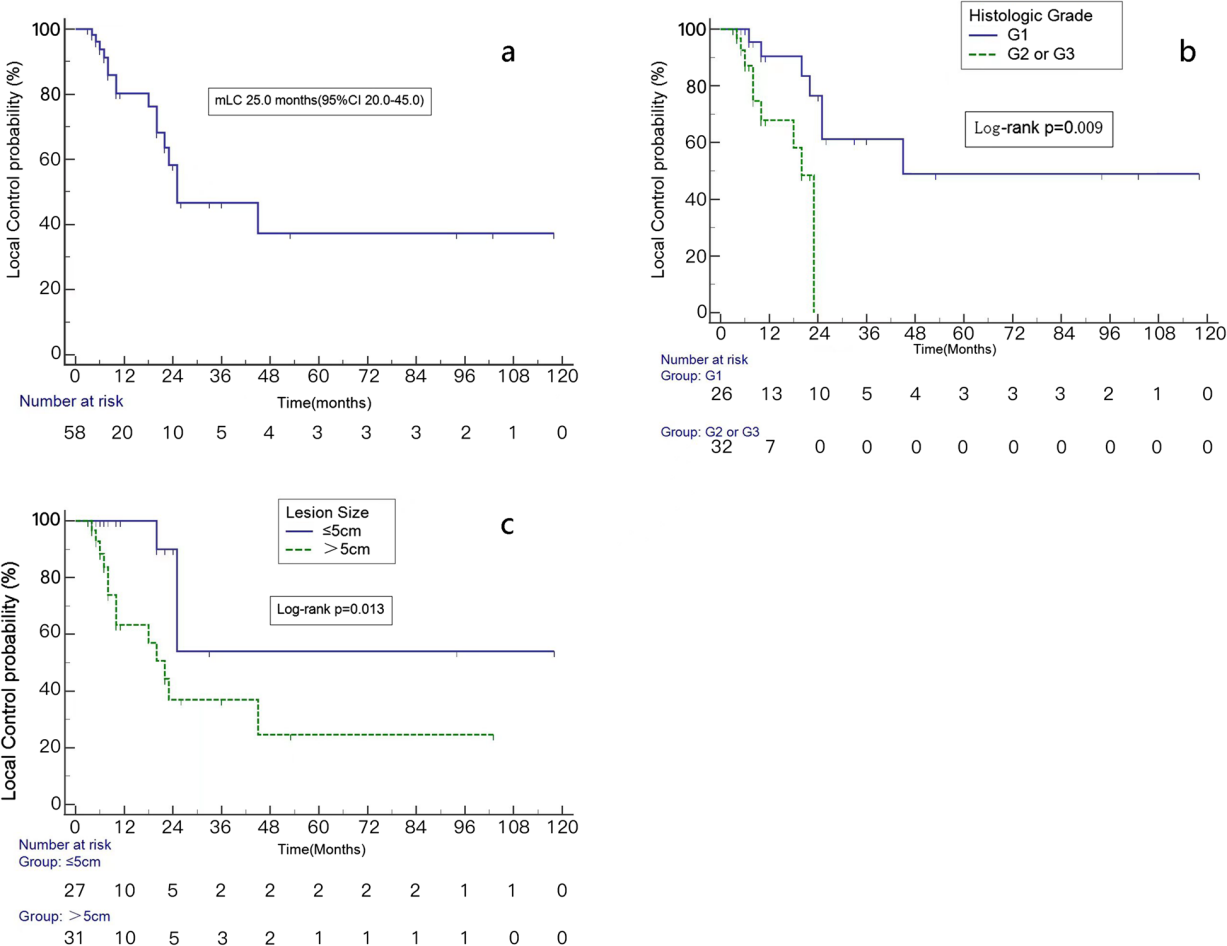
Kaplan–Meier analysis of LC (**a**) and LC of patients with different histologic grade (**b**), lesions size (**c**)

Among the 58 lesions in the 37 patients, the median local control time was 25.0 months (95% CI 20.0–45.0). Overall, the 1-, 2-, and 3-year LC rates were 80.2%, 58.3%, and 46.6%., respectively. The LC of the recurrent and oligometastatic STS is shown in Fig. 2A. In univariate analysis, lesions size (cm) (≤ 5 vs. > 5) and histological grade (G1 vs. G2 and G3) were significantly related to LC (Table 3). Median LC of G1 was 45.0 months (95% CI 22.0–45.0), and the median LC of G2 and G3 was 20.0 months (95% CI 8.0–23.0) (*p* = 0.009) (Fig. 2B). LC with lesions size less than or equal to 5 cm was better (*P* = 0.013) (Fig. 2C). BED did not influence LC (HR = 0.595, *p* = 0.324). Multivariate analysis showed that histological grade, and lesions size also influenced LC (Table 4). It is noteworthy that histological grade was significantly correlated with LC outcomes after adjustment for lesions size, number of lesions and disease setting (recurrent and oligometastatic) (Table 5).

**Table 3:** Univariate analysis for OS, PFS, and LC

	OS		PFS		LC	
	HR 95% CI	*p*	HR 95% CI	*p*	HR 95% CI	*p*
Disease setting	0.363	**0.016**	0.280	**0.003**	0.692	0.495
Recurrence VS oligometastasis	0.159–0.828		0.123–0.641		0.240–1.993	
Histological grade	0.183	**0.0001**	0.179	**0.0001**	0.210	**0.0094**
G1 vs G2 and G3	0.077–0.432		0.074–0.431		0.065–0.682	
Number of lesions^a^	0.335	**0.028**	0.268	**0.009**	0.308	0.056
1 vs 2–3	0.126–0.887		0.099–0.720		0.092–1.030	
Lesions size	0.745	0.483	0.822	0.638	0.282	**0.013**
≤ 5 cm vs > 5 cm	0.327–1.697		0.362–1.863		0.104–0.763	
BED	0.629	0.248	0.597	0.206	0.595	0.324
< 100 Gy vs ≥ 100 Gy	0.278–1.381		0.269–1.327		0.212–1.671	

*Abbreviations:LC* local control, *PFS* progression-free survival, *OS* overall survival, *BED* biologically equivalent dose

^a^Number of concurrent lesions at the time of first SBRT

**Table 4:** Multivariate analysis for OS, PFS, and LC

	OS		PFS		LC	
	HR 95% CI	*p*	HR 95% CI	*p*	HR 95% CI	*p*
Disease setting	0.680	0.488	0.423	0.112	NA	NA
Recurrence vs. oligometastasis	0.230–2.007		0.146–1.223			
Histological grade	0.194	**0.003**	0.193	**0.003**	0.283	**0.045**
G1 vs. G2 and G3	0.066–0.575		0.064–0.581		0.083–0.972	
Number of lesions^a^	1.080	0.893	1.026	0.964	0.313	**0.044**
1 vs. 2–3	0.354–3.300		0.338–3.117		0.102–0.967	
Lesions size	NA	NA	NA	NA	0.210	**0.017**
≤ 5 cm vs. > 5 cm					0.058–0.757	

*LC* local control, *PFS* progression-free survival, *OS* overall survival

^a^Number of concurrent lesions at the time of first SBRT

**Table 5:** Cox regression model showing the HRs for incident LC depending on histological grade

Model	LC	
	HR (95% CI)	*P* value
Model 1: histological grade	0.223(0.065–0.767)	0.017
Model 2: model 1 + lesions size	0.244(0.072–0.831)	0.024
Model 3: model 2 + disease setting + number of lesions	0.283(0.083–0.972)	0.045

### Survival

**Fig. 3:**
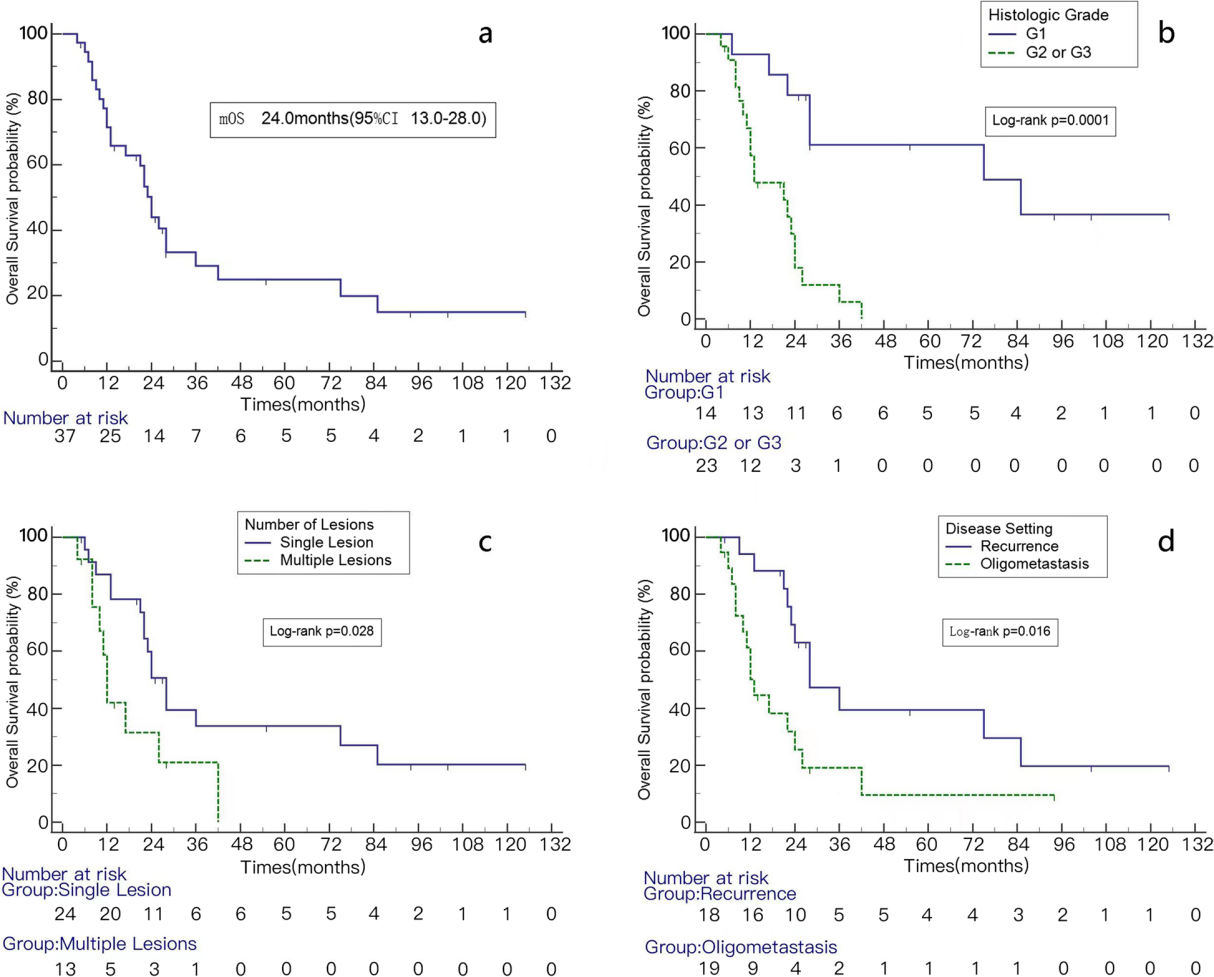
Kaplan–Meier analysis of OS (**a**) and OS of patients with different histologic grade (**b**), number of lesions (**c**), disease setting (**d**)

The median OS was 24 months (95% CI 13.0–28.0 months). The 1-, 2-, and 3-year OS rates were 71.5%, 40.0%, and 29.1%, respectively. A Kaplan-Meier plot for OS is shown in Fig. 3A. Using univariate analysis, BED had no influence on OS and PFS, the following factors were significant prognostic variables for OS: disease setting (*p* = 0.016), histological grade (*p* = 0.0001) and the number of lesions at the time of first SBRT (*p* = 0.028) (Table 3 and Fig. 3B–D). Using multivariable analysis, we know histological grade was an independently significant factor for OS (Table 4). The Median OS of G1 was 75.0 months (95% CI 22.0 to 85.0). One- and 3-year survival rates were 92.9% and 61.1%, respectively. The median OS of G2 and G3 was 13 months (95% CI 10.0–24.0 months), and the 1-year and 3-year survival rates were 57.9% and 6.0%, respectively.

**Fig. 4:**
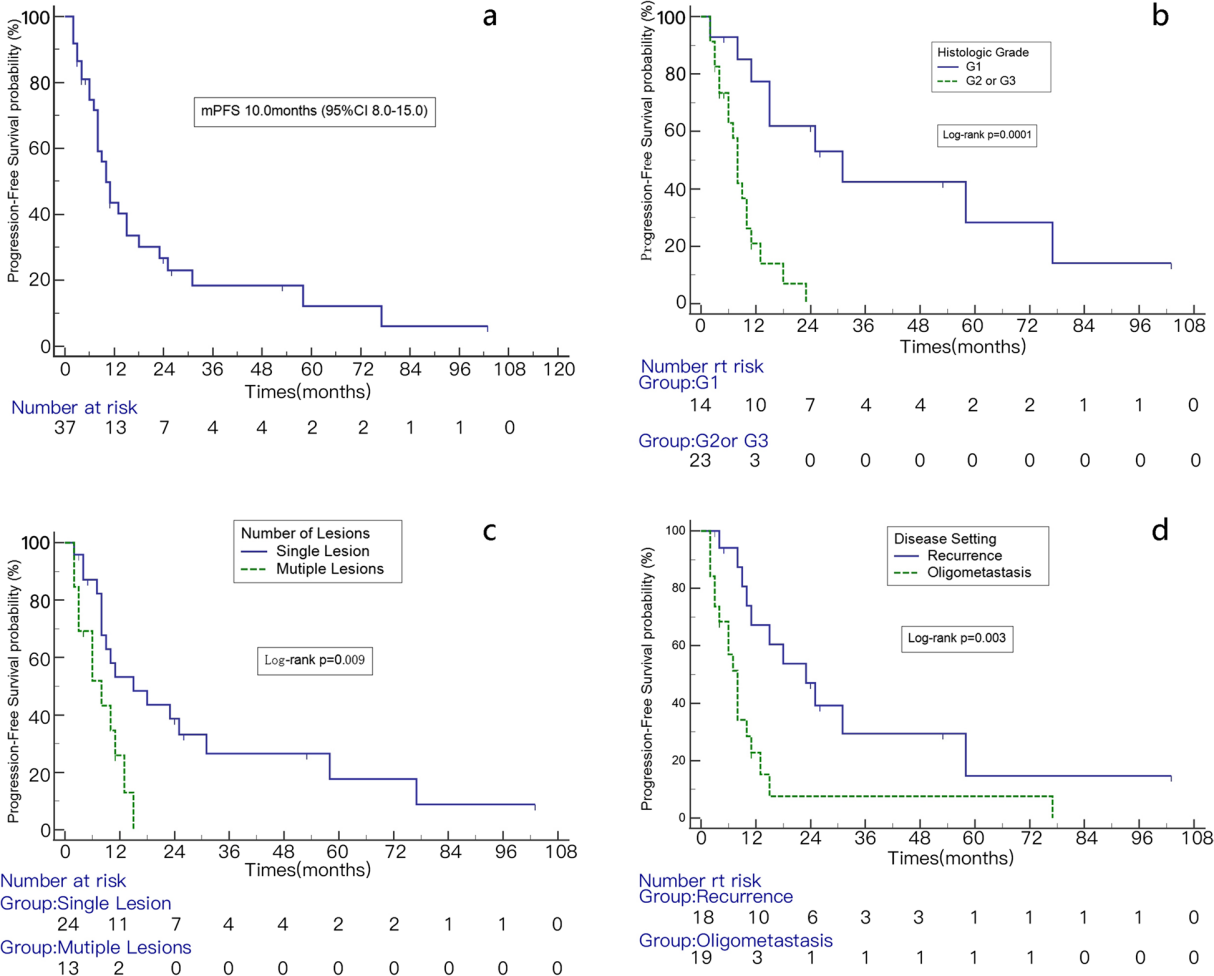
Kaplan–Meier analysis of PFS (**a**) and PFS of patients with different histologic grade (**b**), number of lesions (**c**), disease setting (**d**)

The median PFS was 10.0 months (95% CI 8.0–15.0 months). The 1-, 2-, and 3-year PFS rates were 43.6%, 26.8%, and 18.4%, respectively (Fig. 4A). In univariate analysis, disease setting (*p* = 0.003), histological grade (*p* = 0.0001), and number of lesions at the time of first SBRT (*p* = 0.009) were significant factors for PFS (Table 3 and Fig. 4B–D). In multivariable analysis, the histological grade (*p* = 0.003) was significant factor for PFS (Table 4).

### Toxicity

We found no cases of acute or late grade 4 toxicity or possible treatment-related death. The most common acute toxicity was grades 1–2 fatigue (8/37, 21.6%). Acute grade 2 radiation pneumonitis occurred in one patient (2.7%), and acute grade 1–2 radiation enteritis was observed in two patients (2.7%). Late grade 3 radiation dermatitis was observed in one patient (2.7%) 6 months after SBRT.

## Discussion

STS are rare and heterogeneous mesenchymal neoplasms, with more 70 histological subtypes [15]. 40 to 50% of STS patients will develop metastatic disease [16]. More than 40% of patients underwent more than one surgery because of recurrence. The ability to remove the tumor radically decreased with each recurrence [17]. After multiple surgeries, it is challenging to undergo surgery again in some patients; in such cases, radiotherapy can be an alternative local treatment. We demonstrate that SBRT for recurrent and oligometastatic patients achieved good local control and survival with minimal toxicity. Multivariate analysis showed that histological grade influenced OS、PFS and LC.

SBRT has been increasingly used for patients with tumors that are not candidates for surgery in recent years. For instance, it is now the standard treatment option for early-stage inoperable non-small cell lung cancer due to its superior efficacy [18]. Good results can be achieved not only with SBRT for early-stage tumors but also in patients with advanced oligometastatic [19, 20]. In patients with oligometastatic NSCLC with good systemic control, local treatment of oligometastases (including SBRT and conventional large fractionated radiotherapy) significantly improves PFS and translates into a survival benefit (mOS 41.2 months vs 17 months, *p* = 0.017) [21]. A recent randomized phase II clinical trial compared patients with oligometastatic solid tumors of different histologic types with standard palliative care or no SBRT for all lesions and observed a significant improvement in 5-year OS in the SBRT-treated group (42.3% vs. 17.7%, *p* = 0.001) [19, 20]. In patients with oligometastatic STS (≤ 3 synchronous lesions), SBRT yields satisfying local control with minimal toxicity. Increased time from primary tumor to first metastasis identifies patients with a potentially greater benefit from SBRT [22]. For patients with recurrence and metastasis, several small, single-institution retrospective studies have shown that SBRT has a relatively high local control rate and low toxicity in treating sarcoma pulmonary metastases [7–11]. In response to SBRT versus surgery, C. Tetta et al. [12] study showed that topically applied SBRT for STS lung metastases was associated with lower cumulative overall mortality, and similar overall and disease-free survival compared to surgery. Our study shows that the reduction of local recurrence by SBRT may translate into a survival benefit for patients with oligo-metastases or local recurrence, especially in G1 patients.

Current studies on the role of radiotherapy in STS focused on local recurrences and oligometastatic lesions. Loi M et al. [22] reported 16 oligometastatic patients, with most metastases in the lungs and a few in lymph nodes or soft tissues. The pathological type was mostly liposarcomas, with most (81%) lesions less than 3 cm in size. With SBRT technology, the prescribed dose is 30–60 Gy/1–6 fx, median prescribed EQD2 dose was 115 Gy_10_ (range 60–150). Rates of LC were 84% at 2 years and 78% at 4 years. On univariate analysis, only the first relapse within 24 months was significantly correlated with reduced local control (*p* = 0.022), and no mention of the impact of EQD2 on LC. Stragliotto CL et al. [23] reported that the best response was significantly correlated with the mean dose ofCTV in EQD2 (*p* = 0.018). The study includes STS and other osteosarcomas, all in patients with metastases, including oligometastases and multiple metastases. The most frequent pathological staging in STS was leiomyosarcoma, with lung metastases being the most common (97/136), and OS, PFS, and LC results were comparable to this study. Moureau-Zabotto et al. reported on 83 patients with first local recurrences, of whom 38 patients received surgery alone, 25 surgery and additional RT without prior RT, and 20 surgery with reirradiation mainly via brachytherapy to cumulative doses of 95–115 Gy. Local control after a median follow-up of 59 months was significantly improved by additional RT (64% vs. 45%) with an overall 5-year OS of 54% [24]. For recurrent or oligometastatic soft tissue sarcoma, BED ≥ 100 Gy did not increase the LC in our findings, probably related to greater tumor heterogeneity, but the median BED of our findings was 85.5 Gy, which was higher than external beam radiation therapy (EBRT). SBRT is currently being also investigated for primary tumors. For example, the University of Wisconsin is currently performing a phase II study using SBRT to 60 Gy in 3–8 fractions, including suitable locally advanced and inoperable primary STS (NCT03972930).

Based on common sense speculation, the relevant factors affecting the efficacy of SBRT may be tumor size, prescribed dose, and histology. The prognosis of STS is inferred by the interaction of multiple factors, including varying characteristics of the patient, varying characteristics of tumorigenesis and presentation and specificity of the different modalities, sequences, and combinations of combination therapy [25, 26]. The single-factor analysis or even a multifactor analysis, evaluated in several reports, reveals the impact of the bias of the selection criteria of the patient and the limitation of the sample size. In our study, patients’ OS and PFS were not related to BED, but it is associated with the tumor grade at the time of first treatment. In the regression analysis for LC, multivariate analysis was related to the histological grade, lesions size, and disease setting, but not BED. We maybe infer that the prognosis of STS is related to the characteristics of the tumor itself rather than the characteristics of the treatment.

There are several limitations to the current study. First, the number of cases in this study is small. Second, we did not analyze the effect of different pathological types on local control. Moreover, as a retrospective study of patients treated over a long period of time, selection bias cannot be excluded. Due to the rarity of the disease, a multicenter, clinically controlled study is necessary.

## Conclusion

SBRT is a safe and effective treatment for recurrent and oligometastatic STS and may be a promising treatment strategy for patients who are difficult to operate for various reasons. Patients with histologic G1 may have better local control and survival than those with histologic G2 and G3. Further research in multicenter prospective studies is needed.

## Data Availability

All data generated or analyzed during this study are included in this published article [and its supplementary information files].

## Abbreviations

BED: Biologically effective dose
CI: Conformity index
CT: Computed tomography
GTV: Gross tumor volume
HI: Homogeneity index
LC: Local control
LF: Local failure
MTS: Metastasectomy
MRI: Magnetic resonance imaging
nCI: New conformity index
OS: Overall survival
PFS: Progression-free survival
PTV: Planned target volume
STS: Soft tissue sarcoma
SBRT: Stereotactic body radiotherapy
